# Acknowledgment to Reviewers of *Biology* in 2020

**DOI:** 10.3390/biology10020088

**Published:** 2021-01-25

**Authors:** Biology Editorial Office

**Affiliations:** MDPI AG, St. Alban-Anlage 66, 4052 Basel, Switzerland

Peer review is the driving force of journal development and reviewers are gatekeepers who ensure that *Biology* maintains its standards for the high quality of its published papers. Thanks to the cooperation of our reviewers, in 2020, the median time to the first decision was 18 days and the median time to publication was 37 days. The editors would like to express their sincere gratitude to the following reviewers for their precious time and dedication, regardless of whether the papers were finally published:
Abbott, Jessica K.Licon-Cano, CarmenAbdeljawad, T.Liesveld, JaneAbe, TatsuyaLin, Cheng-WenAbuillan, WasimLin, Chih-LiAdegboye, OyelolaLin, Liang-tingAdur, MalavikaLinder, BenediktAdvani, VivekLindqvist, JohanAgarwal, PoonamLinial, MichalAgüero-Chapin, GuillerminLipscomb, Michael W.Ahmadi, SohaListenberger, LauraAhmed-Braimah, YasirLiu, XiaoleiAlberti, FabrizioLiu, ZhihuaAlexander, Brenda M.Lo, ChristopherAlexis, FrankLondon, EdraAl-Fatimi, MohamedLopatkin, Allison J.Ali, Hossam El-SheikhLopera-Barrero, Nelson MauricioAlipieva, KalinaLopes, Alberto Jorge OliveiraAljabali, Alaa A. A.Losada, AnaAllen, ClaireLotti, Lavinia VittoriaAllen, Lee-Ann H.Loveridge, JoelAlvarez-Narvaez, SonsirayLu, Jeng-WeiAlyass, AkramLu, Pei-LuenAmirkhani, MasoumeLu, Qing-BinAmler, EvzenLualdi, MartaAmpatzis, KonstantinosLuo, BingAmpuero, SilviaLupo, Karen D.Andrade Cetto, AdolfoLusczek, Elizabeth R.Anton, LennikovLuvisi, AndreaAnver, ShajahanLuzardo-Ocampo, IvanAraldi, ElisaMa, ChenAramendia, IñigoMa, HongArango Duque, GuillermoMa, YifanArasanz Esteban, HugoMachado, Maria De Fátima P.S.Aremu, Adeyemi OladapoMacias-Diaz, Jorge E.Argentiero, AntonellaMagdanz, VeronikaArmant, OlivierMage, Rose G.Aromolaran, Ademuyiwa S.Magori, KrisztianArora, HimanshuMahadevan, PadmanabhanArrebola, Francisco A.Majali-Martinez, AlejandroAshley, RyanMaksimov, Igor V.Attia, JohnMaksymiuk, Andrew W.Attisano, LilianaMandala, AshokAugenlicht, Leonard H.Manferdini, CristinaBagheri, ZahraMannino, GiuseppeBagnoli, BrunoManuel Giorgi, FedericoBailly, BenjaminManzu, Ciprian ClaudiuBaird, Sarah K.Mao, DaganBajoghli, BaubakMarasca, ClaudioBajwa, Vikramjit S.Marble, S. ChristopherBaker, Josh E.Marchev, AndreyBaker, Robert L.Marcia, MarcoBakovic, MaricaMarcovici, GenoBalasubramanian, NarayanaganeshMarin Gomez, Oscar H.Balland, EglantineMariottini, PaoloBallini, AndreaMarkopoulos, Georgios S.Bang, HyoweonMarkov, GabrielBarasch, AndreiMarques, Mário António CardosoBarbayannis, NikosMarsh, JustinBarbieri, NicolleMartikainen, RiikkaBarik, SailenMartinez-Pastor, FelipeBarnea, Eytan R.Martinković, FranjoBarral, DuarteMartins, Mariana RenovatoBarrino, FedericoMarzano, Shin-YiBarsyte-Lovejoy, DaliaMarzo, TizianoBartocci, PietroMascitti, MarcoBartošová-Sojková, PavlaMastino, AntonioBartosz, GrzegorzMastrantuono, LucianaBarucca, MarcoMasui, KentaBar-Ziv, RazMatsuo, RyotaBasak, Saroj K.Mattison, Chris P.Bastos, MargaridaMazur-Bialy, AgnieszkaBathula, Chandra SekharMbita, ZukileBatie, MichaelMcCollum, DanBattaglia, FortunatoMcCoy, AnnetteBaudier, Kaitlin M.McDaneld, Tara G.Becker, PeterMcFarlane, Donald A.Bednarek, PiotrMcFarlane, JamesBehem, Christoph R.McMichael, Maureen A.Belaidi, Abdel AliMehterov, NikolayBellardita, CarmeloMelin, FredericBelluzzi, ElisaMelters, DanielBen Kaab, SofieneMenale, CiroBenakanakere, Manjunatha R.Mendelson, AsherBen-Arieh, DavidMerimanov, SerikBenedetti, FrancescaMerrin, JackBennato, FrancescaMezzetti, MatteoBerbert, JulianaMichot, BenoitBerger, JosefMikaelyan, Arsen S.Berillis, PanagiotisMilosavljević, IvanBernotienė, RasaMinervino, Antonio Humberto HamadBertelloni, FabrizioMishra, RosalinBertocchi, MartinaMitrofanova, AllaBeszteri, SaraModesto, PaolaBettaieb, AliMogren, Christina L.Beutner, GiselaMohamed, Azza H.BEZU, LucilliaMolina-Cerrillo, JavierBhat, KamakotiMolnar, LaszlóBhattacharya, AbhisekMoniuszko, AnnaBhave, DevayaniMonteiro, Joana F.Bi, Andy ChengfengMonteiro, LuisBiagi, MarcoMontoya, AnaBillat, Véronique LouiseMoore, Matthew D.Bilski, JanMoore, TaraBiondo, Luana AmorimMorales-González, José AntonioBittel, Adam J.Morán-Diez, María E.Bittel, Daniel C.Morcia, CaterinaBlando, FedericaMoretti, SamueleBlomme, JonasMoriarty, TerenceBobek, JanMoriyama, YasukoBodea, Liviu-GabrielMoro, LoredanaBonamore, AlessandraMortensen, Luke J.Bond, Simon T.Moser, SimoneBonga, Sjoerd Evert WendelaarMouradov, AidynBongarzone, ItaliaMuchembled, JeromeBorda-de-água, LuísMuchero, WellingtonBordes, María Dolores LlobatMuhammad, NaoshadBorsuk, GrzegorzMulo, PaulaBosma, Roel H.Munch, Jean CharlesBossaller, JennyMunoz-Pacheco, Jesus M.Botton, ThomasMurillo-Saich, Jessica D.Botwright, NatashaMurphy, RonanBotzenhart, UteMusacchia, FrancescoBoucher, DaveMutiga, Samuel K.Bourette, Roland P.Myc, AndrzejBowsher, Julia H.Naafs, BernardBoyd, Jonathan W.Nagafuchi, SeihoBrack, AndréNagaraj, ViniBrady, Graham F.Nagata, YosukeBragazzi, Nicola LuigiNagy, IstvanBraig, SimoneNahvi, AliBråkenhielm, EbbaNair, SreenathBrandt, CurtisNaka, HiroakiBrenowitz, Eliot A.Nakabayashi, KazumiBrogden, GrahamNakahata, ShingoBrown, Euan R.Nakai, KeiBruce, Timothy J.Nakajima, KeiBruggmann, PhilipNakamura, GilbertoBrundage, AdrienneNakonieczny, DamianBrundage, Cord M.Nanda, SatyabrataBrunetti, LuigiNanno, MasanobuBuchaim, Rogério LeoneNasierowska-Guttmejer, AnnaBuda, Valentina OanaNatarajan, SivaramanBudachetri, KhemrajNavarro, EstanisBudday, SilviaNaviaux, RobertBulk, EtmarNeiva, Henrique P.Bull, LeonardNelson, Fred R. T.Bungartz, GerdNemoto, WataruBurrola-Aguilar, CristinaNeumann, ArianeBustos, FranciscoNewson, LisaButtigieg, Sandra C.Newton, JasonByadgi, Omkar VijayNgen, Ethel J.Cabaro, SerenaNiederseer, DavidCabrera Orefice, AlfredoNigro, ValentinaCabri, JanNirala, Niraj K.Cacciani, NicolaNishihara, HidenoriCacciola, FrancescoNissilä, EijaCadefau Surroca, Joan AureliNisticò, StevenCaeiro, Maria FilomenaNitta, TakayukiCagin, UmutNivedita, NiveditaCameron, AngusNogales, AmaiaCampa, Carlo CosimoNogalski, MaciejCampisi, GiuseppinaNoge, KojiCampos, Maria Jorge GeraldesNorman, TrevorCandiani, SimonaNorton, Kerri-AnnCappai, Maria GraziaNumakawa, TadahiroCappelletti, VeraNunes, Francis Morais FrancoCardoso, SusanaNuthikattu, SaivageethiCare, AndrewNydam, MarieCarvalho, EdmundNyman, DagCaseys, CélineOberemok, Volodymyr V.Caspari, ThomasObermayer, AstridCastillo Carrion, MercedesOgunjirin, AdebowaleCastillo, PabloOhbuchi, ToyoakiCastro, Mariana S.Ohishi, TomokazuCastrucci, Ana MariaOhshima, TakayukiCatapano, CarloOkada, HiroshiCavacas, Maria AlziraOkada, KohkiCeccatto, Vânia MarilandeOkamura, TakuroCecconi, SandraOk-Seon, KwonCentner, TerryOlaf Koppang, ErlingCermeño, PedroOloso, Nurudeen OlalekanČerný, MartinOno, TakashiChardon, FabienOnukwufor, John O.Chauhan, HarshOpiela, JolantaChecchi, VittorioOrd, KeithChen, ChiderOrtega, Angel L.Chen, HongquanOrtega, FelipeChen, LiangOtero, CarolinaChen, QiPaci, MaurizioChen, Shih-ChiehPaeshuyse, JanChen, XiaowuPage, MelissaChen, Yun-JuPalanisamy, SatheeshkumarCheng, Juei-TangPalla, FrancoCheng, ShixiongPallotta, IsabellaCherkaoui Malki, MustaphaPalmer, ChristopherChevrette, Marc G.Panaccione, DanielChi, GeraldPang, BoChiariotti, PaoloPanickar, KiranChilton, LisaPark, HyunChina, ArnabPark, Shin-HyungChitcholtan, KennyParrino, VincenzoChobot, VladimírPasin, FabioChong, JasminePassos, MarisaChorostecki, UcielPatel, NilkanthChoudhuri, AvikPatini, RomeoChrysanthopoulou, AkriviPaudel, Keshav RajChuang, Hung-YiPecori, RiccardoChuang, Yao LiPerche, FédéricoChung, Byung MinPereira, Olívia R.Ciacci, CaterinaPereira-Lorenzo, SantiagoCilia, GiovanniPereiro, PatriciaCione, ErikaPerestrelo, RosaCiribilli, YariPérez Puyana, Víctor ManuelCłapa, TomaszPerez, FranciscoCluzeau, ThomasPerisanidis, ChristosColavito, Sierra A.Perron, HervéColla, EmanuelaPetillon, JulienColosi, Ioana AlinaPezone, AntonioConesa Zamora, PabloPham, Anh TungConn, David BrucePiconi, CorradoContaldo, MariaPietr, StanisławContreras-Cornejo, Hexon AngelPinart, ElisabethCooper, Arthur J. L.Pinto, CarmineCooper, BretPiquereau, JérômeCornelius, DenisePlachetzki, David C.Cossignani, LinaPlatta, HaraldCosta, Ana RitaPolo, Miguel GómezCosta, DianaPomilio, FrancescoCosta, JoanaPonte, GiovannaCostantini, EricaPonz, FernandoCosta-Pinto, Ana RitaPop, Laura AncuţaCotta, Renato MachadoPorceddu, MarcoCrabbe, John C.Prado-Álvarez, MaríaCrocini, ClaudiaPrasad, AnkushCunha-Reis, DianaPrice-Carter, MarianCyplik, PawełPrieto, Jose M.D’Acquisto, FulvioPrist, Paula RibeiroDagur, Raghubendra S.Pritzker, KennethDavari, MehdiPugliese, AndreaDavidson, IritPulcini, DomitillaDaza-Navarro, PaulaPun, Chi-ManDe Angelis, ElisabettaQanud, KhaledDe Araujo, ElvinQin, HongminDe Bellis, LuigiRagazzi, EugenioDe Berardis, DomenicoRageul, JulieDe Castro, GabrielaRaghavan, SudhirDe Chiara, FrancescoRahimi, NaderDe Clerck, CarolineRahman, AzizurDe Jesus Pereira Godinho Velada, IsabelRahman, Md. AtikurDe La Garza, MireyaRai, VineetaDe La Sen, ManuelRaj, KenDe La Varga, HerminiaRajamohan, ArunDe Mattei, MonicaRajpurohit, SurendraDe Sousa-Coelho, Ana LuísaRamesh, Sunita A.Dealwis, Chris G.Ramos, Marco A.Decaro, NicolaRampanelli, ElenaDelaye, LuisRanawade, AyushDelcea, MihaelaRandi, EttoreDelGiorno, Kathleen E.Ravalli, SilviaDemongeot, JacquesRavegnini, GloriaDemyanets, SvitlanaRazvan-Cosmin, PetcaDeng, PengReinartz, SilkeDenoyer, DelphineReinhard, JacquelineDeshmukh, RupeshReiso, HaraldDeutsch, AlexanderRemón, JordiDeutsch, Stephen I.Ren, LinzhuDey, PriyankarReynoird, NicolasDi Liegro, ItaliaRibeiro Silva, ElisabeteDi Martino, CatelloRibeiro, MiguelDi Micco, SimoneRicchiuto, PieroDiaz, ArielRicci, GiuseppeDiaz, GiselaRiccò, MatteoDiekman, BrianRice, Charles D.Dimayuga, PaulRichardson, Annette C.Dimitriadis, StavrosRiffelmacher, ThomasDimri, ManaliRilcheva, Violeta S.Dina, JuliaRivas, Mariella O.Ding, LinRizzarelli, EnricoDireito, RosaRizzoti, KarineDo Paço Teixeira, AnaRoberts, TerryDo Paço, AnaRobertson, Deborah L.Doddapattar, PrakashRocca, CarmineDolgova, OlgaRodríguez Martínez, MaríaDomalik-Pyzik, PatrycjaRodríguez, Armando AlexeiDomenichini, AliceRodríguez, Matías MorínDomingue, MichaelRoma, CristinDong, LihuaRomiti, Giulio FrancescoDonohue, TerrenceRondon, AurélieDonze-Reiner, TeresaRoques, LionelDöppner, Thorsten R.Rosenberg, MosheDrescher, Hannah K.Rosenzweig, AllisonDugé De Bernonville, ThomasRossi, RiccardoDuran, Sue HudsonRotondo, John CahrlesDuran-Frigola, MiquelRout, BhimsenDuryee, Michael J.Rouyer, FrancoisDzieciol, MonikaRoy Chowdhury, SandipanDziegielewska, BarbaraRuban, DmitryDziekońska, AnnaRubinowska, KatarzynaEckel-Mahan, Kristin L.Ruiz-Ruiz, CarmenEdamakanti, ChandrakanthRusu, AdrianaEhrhardt, MatthiasRuzicka, James J.Eid, NabilRytelewski, MateuszEisenbach, MichaelSadasivam, MohanrajEl Bounkari, OmarSadoul, BastienEl Ghoch, MarwanSafavi-Naeini, MitraEl-Aarag, Bishoy Y.A.Safi, CarlEldridge, SandySaito, KosukeElghobashi-Meinhardt, NadiaSaito, SatoshiElla, BhagyarajSaitoh, KenjiEller, FredSala, ClaudiaElmouki, IliasSalvaggio, AntonioElžbieta, DumnickaSamsoondar, JoshuaEmbers, MonicaSanchez Lasheras, FernandoEmmert, HilaSanchez-Martinez, MelchorErséus, ChristerSánchez-Mendoza, Eduardo H.Escalona, Hermes E.Sánchez-Pérez, Ana MaríaEspenberg, MikkSancho, JaimeEspinosa, María Del Rosario MoralesSanda, MiloslavEsteves, Ana CristinaSándor, Attila D.Esumi, TomoyaSantos, CarlaEvangelopoulos, MichaelSantos, Jose MariaEvans, PatriciaSantos-Jimenez, ZurisadayFaccio, GretaSarasija, ShaarikaFaísca, Patrícia F. N.Saretzki, GabrieleFalchetti, Maria LauraSarriés, María VictoriaFalsini, SaraSartorelli, PatriciaFaria, Jorge M. S.Sasaki, Sergio DaishiFassl, AnneSathishraj, RajendranFaulkes, ZenSauerwein, WolfgangFavero, GaiaSavoi, StefaniaFeng, HuapengSaxena, VikasFeresin, GabrielaSchardl, Christopher L.Ferguson, Matthew L.Scharf, MichaelFernando, Eustace Y.Scheibye-Knudsen, MortenFerrari, PaolaScherthan, HarryFerrater, JedelizaSchluepmann, HenrietteFerreira, Leonardo M.R.Schlumbaum, AngelaFerris, TimothySchmitt, Clemens A.Fialho, JoãoSchneider, SimonFidalgo, MiguelSchoefs, BenoîtFilippone, EdgardoSedlock, Jodi L.Fisher, Jonathan S.Seeherunvong, TossapornFlisikowski, KrzysztofSemova, IvanaFogacci, FedericaSene, NdolaneFois, MauroSengupta, SonaliFont, RafaelSequeda-Castañeda, Luis G.Fonte, PedroSerra-Cobo, JordiForlano, RobertaSerrano, MarioForoud, NoraSerre-Beinier, VéroniqueFostier, JanSertorio, MathieuFowden, AbigailSethi, GautamFramenau, VolkerShackelford, JuliaFrancesco, RanaldiShafiq, SarfrazFrancesco, SmedileShah, RaviFranco, J. AgustinShamieh, SaidFregnan, FedericaSharma, AnchalFu, BeideShelton, DeliaFu, WeiqiSheng, KuanweiFukunaga, TsukasaSheshadri, NamrathaFukushima, AtsushiShiba, KiyotakaGad, AhmedShigeno, ShuichiGadea, JoaquinShimizu, YujiGahr, ManfredShin, Ok SarahGaiotti, FedericaShirole, NitinGala, Rikhav P.Shmakov, Sergey A.Galelli, LucaShojaei, ShahlaGalstyan, AnahitShuker, DavidGambardella, MarinaShukla, ArvindGanji, RakeshSiddappa, ByrareddyGarcia, Brandon L.Sidoti, AntoninaGarcia, JoseSienkiewicz, ZenonGarcía-Barrado, María JoséSilas Fernandes, EtoGarcia-Rubio, RocioSilva, CatarinaGardikis, KonstantinosSilva, JoãoGašić, UrošSimkin, JenniferGastineau, RomainSimões, Marta FilipaGates, ColinSingh, NeenuGault, VictorSingh, ParamGawel, DamianSinjari, BrunaGecheva, GanaSivaev, Igor B.Gelinas, AmySivanesan, IyyakkannuGentile, CarlaSkorik, Yury A.Gentili, RodolfoSkrinjar, IvanaGeoffroy, Cédric G.Smith, AnnGerbeau-Pissot, PatriciaSmith, Doug W.Ghidini, MicheleSmith, MarkGhosh, AjitSmith, RobertGiacometti, AndreaSoderhall, IreneGianfredi, VincenzaSoliño, LucíaGianturco, LuigiSolitro, Giovanni FrancescoGibbons, GarrettSon, Tae YunGiordani, BarbaraSong, JoeGiuffrè, MauroSosna, JustynaGiuliani, MaurizioSouza Jr., M. T.Gkika, DimitraSpady, BlakeGoda, TadahiroSpartalis, EleftheriosGomes, NelsonSpiegel, MartinGómez Castilla, JordiSpletter, MariaGómez, Juan Carlos CastroSrimathveeravalli, GovindGomez-Moracho, TamaraStacchiotti, AlessandraGómez-Sánchez, RubénStallsmith, BruceGómez-Zorita, SaioaStanford, StephanieGonzalez, GabrielSteels, ElizabethGonzalez-Parra, GilbertoStein, Derek R.Gopalakrishnan, GopakumarSteward, RobertGordon, KacyStoian, Anca Mihaela PanteaGorgoglione, BartolomeoStrekalova, TatyanaGorshkov, KirillStricker, RaphaelGouveia, Maria JoãoStubbs, Christopher J.Grabinska, KarionaSturtevant, DrewGrant, MichaelStushnoff, CecilGreer, DennisStyrsky, John D.Greischar, Megan A.Subramaniam, DharmalingamGrimaldo, EduardoSuddala, Krishna ChaitanyaGrinde, Maria T.Suenaga, HikaruGruber, AnsgarSugimura, HaruhikoGrzesiak, JakubSun, FengjieGuallar, DianaSun, XuejunGuevara González, RamónSun, YingGuiard, BrunoSung, I-HsinGültas, MehmetSurguchov, AndreiGumieniczek, AnnaSurmacz, LilianaGuo, YanSuzuki, MinoruGupta, DevendraSuzuki, TakashiGyselaers, WilfriedSuzutani, TatsuoHabas, KhaledSvenningsen, PerHall, Steven G.Svitkin, YuriHan, DongSyeda, AishaHan, HongyunSyed-Abdul, Majid MufaqamHanaka, AgnieszkaSzetpizeowski, JacekHanba, Yuko T.Szewczyk-Golec, KarolinaHanchate, NareshSzondy, ZsuzsaHardesty, JosiahSzot-Karpińska, KatarzynaHardtke-Wolenski, MatthiasSzweda, PiotrHardwick, James P.Tabacchioni, SilviaHarke, MatthewTailhades, JulienHarrel, Stephen K.Tam, Samantha H.Hartig, AndreasTamariz, ElisaHayashi, RyujiTan, LiHayes, Sidney J.Tanaka, MasaruHe, ZhongqiTanemura, KentaroHeeke, SimonTang, JijunHegde, ShivanandTang, KaiHelfer, StephanTanini, MarcoHelmy, Yosra A.Tao, LimingHenderson, GreggTashima, ToshihikoHenderson, Michael X.Tatischeff, IrèneHenker, ChristianTaub, MaryHernandez, IgnacioTaylor, JaniceHernot, SophieTchórzewska, DorotaHertel, MoritzTedim, Ana P.Hikichi, YasufumiTellier, MichaelHildreth, Blake E.Templeton, Matthew D.Hill, CameronTerkawi, Mohamad AlaaHinrichs, HermannTerzi, ValeriaHirata, YokoThalamuthu, AnbupalamHjelmen, Carl E.Thilmony, RogerHo, Cheng-MawThirumalaikumar, Venkatesh PeriyakavanamHo, Jar-YiThompson, Miles D.Ho, MengfeiTimperio, Anna MariaHofman, CourtneyTiniakou, EleniHohmann, TimTochkov, KirilHolert, JohannesTognini, PaolaHonrao, ChandrashekharTokas, TheodorosHoole, DavidToriba, TaiyoHorn, IvoTosha, TakehikoHorník, MiroslavTotta, PierangelaHoshide, Aaron K.Toyomizu, MasaakiHoste, HerveTrecroci, AthosHotea, IonelaTrifunovic, DraganaHowes, Laurence GuyTriviño-Tarradas, PaulaHruz, Paul W.Troncone, GiancarloHsu, Te-HuaTroschel, FabianHu, YuanTrucillo, PaoloHuang, LeiTrzaskowska, MonikaHuber, Roland G.Tsai, Kun-LingHund, MartinTsang, MichaelHunter, ElizabethTsuji, TakashiHuntosova, VeronikaTsukahara, ToshifumiHynčík, LuděkTurkiewicz, Igor PiotrIannelli, Maria AdelaideTzang, Bor-ShowIatsenko, IgorUchimiya, MarioIkuta, NaokoUimari, AnneIldikó, TarUrra, José MiguelImani, MahdiUsui, TadaoImpellizzeri, DanielaVaamonde, CarlosInoue, HirofumiVacante, MarcoIrving, TomVaiman, DanielIshii, KenichiValenzuela, Juan LuisIslam, Mohammad ShahidValenzuela, RodrigoIssaeva, NataliaVan Der Blom, JanIssop, LeeyahVan Muiswinkel, Willem B.Ivanova, LarissaVarga, MátéIwata, HisatakaVázquez, María DoloresIzasa, HisashiVeeravalli, VijayabhaskarJahandideh, ForoughVelegzhaninov, Ilya O.Jain, EraVenditti, VincenzoJakubowska, MonikaVenkataraman, ShrinivasJakus, ZoltanVera Reyes, IleanaJana, BimalVerket, AndersJantsch-Plunger, MichaelVicente, ClaudiaJaradat, AbdullahVieira, MónicaJay-Allemand, ChristianVignini, AriannaJenei-Lanzl, ZsuzsaViswanathan, SowmyaJenne, Craig N.Vitale, LucaJeong, Ho-ChangVitale, Salvatore GiovanniJeremic, Aleksandar M.Vitali, AndreaJiang, Yan-BinVitali, MatteoJiménez Rojo, LucíaVives Cobo, CristinaJimenez, JuanVivès, CorinneJnawali, KamalVivien, RégisJohansen, Matt D.Volkovitsh, Mark G.Johnson, James ChadwickVolpicella, MariateresaJong, Chian JuVon Kobbe, CayetanoJose, MarcoVuko, ElmaJoshi, AmitWagner, Hanna JeannineJoshi, Pushpa RajWakabayashi, KatsuzoJourdain, AlexisWalker, Robert P.Jucker, CostanzaWallis, Graham P.Julian, LisaWalyaro, D. J.Kaether, ChristophWan, ShibiaoKalmár, ZsuzsaWang, BoKaneko, GenWang, ChihueiKang, JonghoonWang, HsiuyingKapischke, MatthiasWang, JianxuKarakaidos, PanagiotisWang, MingmingKarim, RoksanaWang, NingKashaninejad, NavidWang, ShuaishuaiKasman, LauraWang, TaoKasuga, KensakuWang, Tzu-FanKato, TakamitsuWang, WenqiKatsani, KaterinaWang, ZhiqiangKausrud, KyrreWang, ZhuoKazimírová, MariaWard, BessKemper, HanWarren, JimKerbl, AlexandraWatterson, StevenKeren, IdoWautier, Jean-LucKerr, CliffWegwitz, FlorianKhambu, BilonWei, YanqiaoKhan, NababWeiss, AndreaKharche, Sanjay R.Weithoff, GuntramKhoa, Vo AnhWejnerowski, ŁukaszKhosla, AashimaWen, JianjunKim, DeokWen, SijinKim, Gi BeomWharton, Rebekah E.Kim, JongWhelan, NathanKim, Joon-hoonWhipple, Chery A.Kim, Min-HyunWhittington, AndrewKim, Tae MinWicky Collaud, ChantalKim, Yong-IckWijesekera, ThiliniKimura, SeisukeWilliams, Stephen J.Kirgan, Daniel M.Wilson, HoustonKiss, AttilaWilson, JamesKlec, ChristianeWindhorst, SabineKleier, Daniel A.Wong, Clarence T. T.Kleindorfer, SoniaWong, Fung-FuhKoeppe, Roger E.Wong, Te-ChihKoga, FumitakaWong, WingKoh, Jin-HoWood, Jennifer L.Kolberg, Hans-ChristianWood, Scott T.Kolodziej, BarbaraWu, JianhongKomuraiah, MyakalaXanthou, GeorginaKondo, TomoXaplanteri, PanagiotaKonwerski, SzymonXia, TianKorytar, TomasXie, JianboKöster, DariusXin, DongyueKotula-Balak, MałgorzataXu, WenyanKoudounas, KonstantinosXu, XiyanKouokam, Joseph CalvinXue, GuangaiKousoulaki, KaterinaYamamura, SoichiroKovalevsky, AndreyYan, ShanKoziorowska-Gilun, MagdalenaYan, ShengminKraemer, GeorgeYáñez Avendaño, Dionisio F.Kraska, PiotrYang, Chao-YieKrell, Rayda K.Yang, JialeKrishnasamy, GopinathYang, JinghuaKuba, MichaelYang, MelindaKubiak, Jacek Z.Yang, YeKulma, MartinYang, YongkangKumar, AshokYang, ZhihongKumar, DhirendraYaparla, AmulyaKumar, PradeepYeste, MarcKumar, ShaileshYoon, Do-YoungKummer, UrsulaYoshida, NaofumiKunji, Edmund R.S.Young, Colin NealKunnev, DimiterYu, LingKusters, IljaYu, RongminKwartowitz, DavidYu, YangKwiatkowski, PawelYuh, Chiou-HwaLacerda, André Eduardo BiscaiaYumoto, IsaoLagali, PamelaYumura, YasushiLai, YandongZachleder, VilémLange, SigrunZahan, MariusLanoue, JasonZajdel, AlicjaLanzafame, Raymond JZaldibar, BeñatLao, OscarZambrano Mendoza, Maria ClemenciaLatté, Klaus PeterZanza, ChristianLeaver, David J.Zefferino, RobertoLebiedzinska-Arciszewska, MagdalenaZeltner, NadjaLedda, SergioZhan, XianquanLee, Bong HoZhang, Jing-ZeLee, Brenda Y.Zhang, QiuyangLee, Eun-WooZhang, XiaoyuLee, Hsueh-TeZhang, YubinLee, Joo HyoungZhao, QianLee, SeunggyuZhao, WeiLee, Tse-MinZheng, GuopingLee, Tsung-HsienZherebtsov, EvgenyLenartowska, MartaZhou, ChengLes, FranciscoZhu, HuaLescure, AlainZhu, LinLesser, IrisZhuo, ChunliuLewis, James D.Ziats, Nicholas P.Ley, Alexandra C.Zienkiewicz, AgnieszkaLey, KlausZino, LorenzoLi, Po-HsienZucconi, LauraLi, TaoTaoZuellig, RichardLi, WanlongŻukowski, KacperLi, Zhi-yongŽunar, BojanLiagre, BertrandŽupanič, AnžeLiao, ZehuanZurita, Mario

